# Letter: Could endothelial dysfunction and vascular damage contribute to pain, inflammation and post-exertional malaise in individuals with myalgic encephalomyelitis/chronic fatigue syndrome (ME/CFS)?

**DOI:** 10.1186/s12967-022-03244-7

**Published:** 2022-01-24

**Authors:** Jeffrey Lubell

**Affiliations:** Norwich, VT, USA

To the Editor,

In their hypothesis paper, Wirth, Scheibenbogen, and Paul describe how endothelial dysfunction could produce a wide range of neurological symptoms in people with ME/CFS [1]. As they and others work to refine their understanding of ME/CFS and the related Long COVID syndrome, I would encourage consideration of the possibility that endothelial dysfunction and vascular damage could also explain other symptoms, including widespread pain and inflammation and post-exertional malaise.

For the past four years, my wife and I have been caregivers for our teenage daughter, who has ME/CFS, hypermobile Ehlers-Danlos syndrome, craniocervical instability, Chiari malformation and several other comorbid conditions. Through observation and trial and error, I have developed a number of hypotheses on these matters that I offer here in the hope they might prompt formal research into how to effectively treat these conditions [2].

## Widespread pain and inflammation

Discussion of endothelial dysfunction and vascular damage in ME/CFS and Long COVID generally focuses on how leakages from dysfunctional blood vessels lead to reduced blood flow, which has many consequences, including reduced oxygenation of muscles and reduced cerebral brain flow. As researchers study this phenomenon, I would encourage consideration of the additional possibility that the leaking fluid causes independent damage. Lipedema researchers have found that leakages from microangiopathic blood vessels cause an excess of interstitial fluid that stimulates the formation of subcutaneous adipose tissue [3], which generates hypoxic conditions and becomes fibrotic, contributing to pain and inflammation [4].

I hypothesize that a similar process happens when fluid leaks from faulty blood vessels in ME/CFS, possibly exacerbated by endothelial dysfunction in lymphatic vessels that inhibit the fluid’s removal, causing widespread pain and inflammation. This mechanism appears most pronounced among people with hypermobility or other connective tissue disorders, a common trait among people with both ME/CFS and lipedema.

My daughter experiences pain from fibrotic adipose tissue as well as what appears to be nerve compression from accumulated interstitial / lymphatic fluid. Manual lymphatic drainage, the squeezing of affected tissue, and the manual break-up of fibrotic adipose tissue have helped to ameliorate these symptoms.

In my daughter, I have also observed impaired drainage of fluid from the glymphatic system, both at the cribriform plate and down her spine. Could this be related to damaged lymphatic vessels or blockages from fibrotic adipose tissue?

## Post-exertional malaise

Like many people with moderate or severe ME/CFS, my daughter struggles to recover from even small amounts of physical exertion. In addition to mitigating her pain, manual lymphatic drainage and the squeezing of affected tissue greatly accelerates this recovery process. We have observed a direct dose–response relationship: the more exercise, the more fluid is present in her tissues, and the more manual draining / squeezing is necessary for her to recover.

Based on this experience, I hypothesize that excess interstitial fluid resulting from dysfunctional blood and lymphatic vessels contributes to the experience of post-exertional malaise, with fluid literally drowning affected tissue, leading to hypoxic conditions and inflammation. Possible explanations for the increased interstitial fluid are increases in blood pressure during physical exertion, hypermobile joints going out of place, prompting localized increases in interstitial fluid, and increases in cortisol that generate an increase in fluid and blood volume. Increases in fluid leakage due to elevated cortisol levels may also explain why some people with ME/CFS feel worse when stressed or anxious. The role of cortisol (or another mediator with fluid retaining properties) may explain why cognitive exertion can also generate post-exertional malaise. When present, elevated estrogen levels may exacerbate leakage by increasing fluid volume.

I am not sure why there is typically a delay between physical exertion and the experience of the most acute symptoms of post-exertional malaise. One possibility is that it takes time for the tissue inundated with fluid to feel the full effects of the hypoxic conditions. Another possibility is that a biphasic reaction triggered during physical exertion leads to the release of a mediator that causes heightened endothelial dysfunction and fluid release.

Further research is needed into the causes of endothelial dysfunction and damage (in addition to initial infection and inflammatory overreaction, consider major “crashes,” mast cell activations, surgeries and microclots as additional contributors) and appropriate treatment.

## Data Availability

Not applicable.
